# Serum Exosomal lncRNA AC007099.1 Regulates the Expression of Neuropeptide-Related FAP, as a Potential Biomarker for Hepatocarcinogenesis

**DOI:** 10.1155/2022/9501008

**Published:** 2022-02-10

**Authors:** Luqian Li, Fei Xiao, Guolian Liu, Yufeng Chen

**Affiliations:** Department of Clinical Laboratory Medicine Maoming People's Hospital, China

## Abstract

Neuropeptide-associated fibroblast activation protein (FAP) may be an important risk factor for neurovascular metastasis in hepatocellular carcinoma. Analysis of The Cancer Genome Atlas (TCGA) database showed that *FAP* mRNA was highly expressed in most human tumor tissues. The HPA database then verified that *FAP* was highly expressed in tumor tissues following protein translation. Survival analysis then showed that the level of *FAP* expression significantly affected the overall survival (OS), progress free interval (PFI), and disease specific survival (DSS) of patients with hepatocellular carcinoma. A high expression of *FAP* in tumor tissue is associated with poor patient prognosis. According to the results of spearman correlation, AC009099 and *FAP* were negatively correlated with miR-7152 expression, while AC009099 and *FAP* expression were positively correlated. The lncRNA AC007099.1, which may serve as a potential target for the treatment of hepatocellular carcinoma, was associated with liver cancer. AC007099.1/miR-7152/FAP was found to be associated with immune infiltration in patients with hepatocellular carcinoma. Enrichment analysis suggests that the AC009099/miR-7152/FAP ceRNA regulatory network is associated with neuropeptide functional pathways. In conclusion, a neuropeptide-related AC009099/miR-7152/FAP ceRNA regulatory network was constructed in this study.

## 1. Introduction

Liver cancer is one of the deadliest cancers in the world, and 70% to 90% of primary liver cancers are hepatocellular carcinomas. The incidence of the disease is increasing annually, with a higher prevalence in men than in women [1, 2]. At present, there is still a lack of effective treatments for hepatocellular carcinoma, except for physical and chemical treatments. These treatments include radiation, ablation, surgical resection, transplantation, and transcatheter arterial chemoembolization, while only a few expensive drugs have little effect [3, 4]. However, the specific mechanisms underlying the development of hepatocellular carcinoma have not been elucidated and need to be further explored [5].

Tumor microenvironment helps cancer cells evade immune surveillance and supports their proliferation, metastasis, and dissemination [6–8]. The heterogeneity of hepatocellular carcinoma cells then causes drug resistance in hepatocellular carcinoma, which serves as a major challenge in treating hepatocellular carcinoma. The cancer microenvironment is generally stable and rarely progresses to drug-resistant subtypes. Therefore, it is much more favorable to select a component of the microenvironment as an antitumor target than a drug-resistant cancer cell.

Neuropeptides are emerging as therapeutic targets for the prevention of tumor-induced neurogenesis and immune escape [9]. Some studies have shown that various neuropeptides change tumor microenvironment during tumorigenesis, progression, and prognosis [10–12]. Fibroblast activation protein (FAP) is a dipeptidyl peptidase (DPP) and endopeptidase that is weakly expressed in normal adult human tissues but is greatly upregulated in activated mesenchymal cells of tumors [13]. A number of neuropeptides including Neuropeptide Y, B-type natriuretic peptide, substance P, and peptide YY have been found to be hydrolytic substrates of FAP [13]. Neuropeptide-related FAP has a low expression in normal tissues but is elevated in inflammatory conditions [13]. Multiple studies have shown that *FAP* expression level is elevated in esophageal, colorectal, pancreatic, and breast cancers and has a role in promoting cancer progression [14]. We speculate that FAP may be an important risk factor for neurovascular metastasis of hepatoma.

Recent studies have revealed that neuropeptides in a variety of tumor systems may be regulated by exosomes and that abnormalities in such regulation can affect neuroendocrine function [15–17]. Exosomes are membrane-bound vesicles that can transport biologically active molecules between cells [18]. In addition to regulating the microenvironment between cells and the immune system, exosomes also exchange and transmit genetic material in the intracellular space through “transport” [19–21]. In addition, exosomes are involved in the metastasis of tumor cells, angiogenesis, and the transformation of normal cells into tumor cells [22]. Tumor-derived exosomes contain many serological markers associated with cancer, such as miRNAs that can be used to detect early HCC. Exosomes secrete miRNAs and other substances that regulate the growth and metastasis of HCC. For example, the miRNA-21 secreted by HCC cells activates PDK1/AKT signalling in HSC, transforming HSC cells into CAFs. Other factors such as TGF-*β* further contribute to the development of cancer [23]. Furthermore, the binding of Toll-like receptors (TLRs) in immune cells to exosomes containing miRNA-21 and miR-29a activates the NF-*κ*B pathway in TLRs. This signaling cascade leads to the secretion of a series of inflammatory factors that promote tumor growth and metastasis [24]. We propose a hypothesis that neuropeptide FAP in HCC may be regulated by exosomes in the tumor microenvironment.

The development of microarray and RNA sequencing technologies has facilitated further research into RNA and DNA. Such technologies have become an important part of biomedical research [25, 26]. The identification of liver cancer-specific molecular biomarkers further revealed the potential molecular mechanism of liver cancer, which is important for the early diagnosis, prognosis monitoring, and development of new molecular targeting drugs for this disease. In this study, differentially expressed genes (DEGs) of HCC were identified from the TCGA database. The functions and key genes obtained were then systematically analyzed as potential biomarkers during the development and prognosis of hepatocellular carcinoma.

## 2. Materials and Methods

### 2.1. Data Sources and Treatment of Differential Genes

The transcriptome sequencing data of the TCGA liver hepatocellualr carcinoma (LIHC) dataset and the corresponding clinical data were downloaded from The Cancer Genome Atlas (TCGA) database [27]. The downloaded gene expression data and clinical data files were decompressed into text files, and the data was organized into processable expression matrix data using the R software program (version 3.6.3). The human gene annotation file was downloaded from the Ensembl website (http://asia.ensembl.org/index) and converted to Gene ID in R for further analysis. Moreover, the Human Protein Atlas (HPA) (https://www. http://proteinatlas.org/) was used to observe and compare the expression levels of the identified key genes in normal and liver cancer tissues. LNCipedia was used to obtain the sequence of the lncRNA [28]. lncLocator was used to obtain subcellular localization of target lncRNAs [29].

### 2.2. Construction of Exosome-Neuropeptide ceRNA Network for Hepatocellular Carcinoma

DIANA-LncBase (http://www.microrna.gr/LncBase) is a database dedicated to documenting lncRNA-miRNA interactions. It includes all experimentally validated lncRNA-miRNA interactions in the literature. The DIANA-LncBase web tool (http://www.microrna.gr/LncBase) was used to predict potential target miRNAs for exosomal lncRNAs [30]. miRWalk is a comprehensive database of miRNA target genes (http://mirwalk.umm.uni-heidelberg.de/). miRWalk was used to predict potential miRNAs that might bind FAP. It not only records miRNA binding sites on the full-length sequences of genes but also combines them with predictions from 12 existing miRNA target prediction programs (DIANA-microTv4.0, DIANA-microT-CDS, miRanda-rel2010, mirBridge, miRDB4.0, miRmap, miRNAMap, doRiNA i.e., PicTar2, PITA RNA22v2, RNAhybrid2.1, and Targetscan6.2) to predict the binding information set for binding association [31].

### 2.3. Gene Set Enrichment Analysis (GSEA)

GSEA was used to predict the pathway of FAP enrichment in hepatocellular carcinoma by GSEA [32, 33]. Patients with hepatocellular carcinoma were divided into high- and low-expression groups according to the median value of FAP expression. The c2.cp.kegg.v7.1.symbols.gmt functional dataset from MsigDB (The Molecular Signatures Database) was used as the reference gene set, and the normalized microarray matrix was analyzed. The number of random combinations was set to 1000.

### 2.4. Methylation and Expression Analysis

DNA methylation is an epigenetic difference. The human disease methylation databases DiseaseMeth (http://bio-bigdata.hrbmu.edu.cn/diseasemeth/), UALCAN (http://ualcan.path.uab.edu/), and MEXPRESS (https://mexpress.be) were used to assess the correlation between the methylation levels and the expression of FAP in hepatocellular carcinoma and normal tissues adjacent to the carcinoma.

### 2.5. Immune Cell Infiltration Analysis

Analysis of RNA-seq data from different subgroups of patients with liver cancer using the CIBERSORT algorithm was performed to determine the relative proportions of 22 immune infiltrating cells as previous research [34, 35]. Spearman correlation analysis was performed for gene expression and immune cell content. A *p* value < 0.05 was considered a statistically significant difference.

### 2.6. Survival and Statistical Analysis

The results were statistically analyzed using the R software (version 3.6.0). Quantitative data were expressed as mean ± standard deviation, and a *t*-test was used for between-group analysis. Differences in the expression of the characteristic factors in the different groups were assessed using *t*-tests. Survival curves were plotted using the Kaplan-Meier method, and the overall survival time (OS), progression-free survival time (PFS), and disease-specific survival rate (DSS) were analyzed.

## 3. Results

### 3.1. Upregulation and Characterization of FAP Expression in Tumors

**Figure 1:**
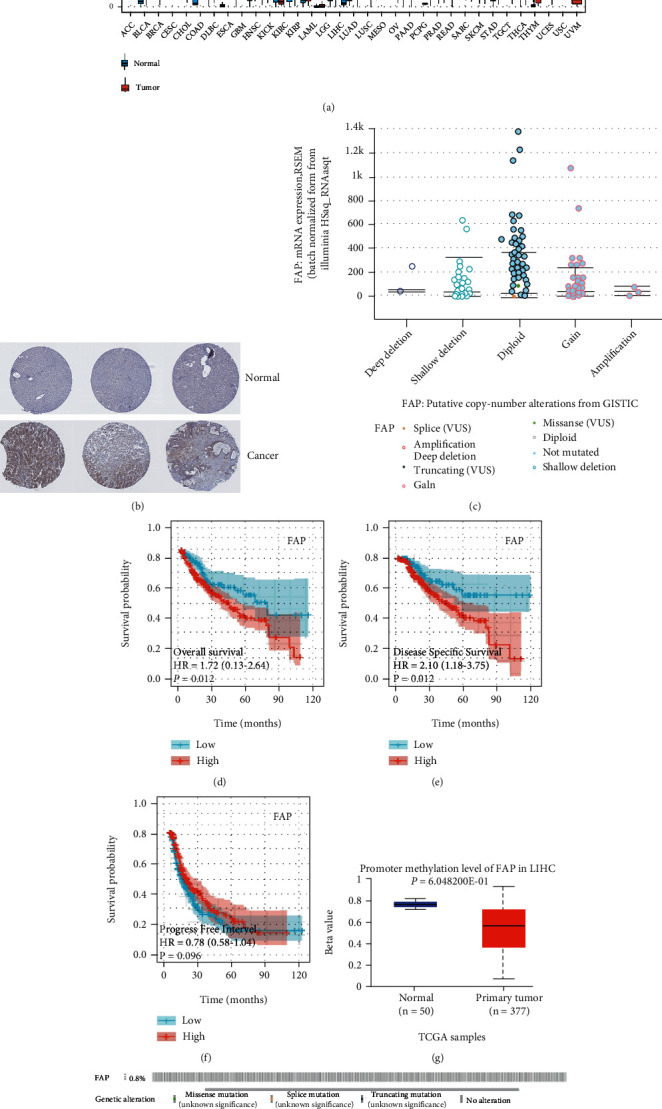
Upregulation and characterization of *FAP* expression in tumors. (a) Characterization of FAP expression in pancytopenia. (b) Validation of *FAP* expression at the translational level by the HPA database (immunohistochemistry). (c) *FAP* copy number differences in TCGA-LIHC. (d–f) The correlation of survival time (OS) (d), progression-free survival (PFS) (e), and disease-specific survival (DSS) (f) with *FAP* expression was analyzed by Kaplan-Meier method for plotting survival curves, respectively. (g) Differences in *FAP* promoter methylation in normal and tumor groups of TCGA-LIHC. (h) cBioPortal-based demonstration of genomic alterations of *FAP* in TCGA-LIHC.

TCGA database analysis shows that the FAP gene is highly expressed in most human tumor tissues (Figure 1(a)). The HAP database verified the expression of FAP at the translational level and found that the protein was highly expressed in tumor tissues (Figure 1(b)). There was a significant difference in the FAP copy number profile in TCGA-LIHC (Figure 1(c)). Results of the survival analysis showed that the expression levels of the FAP gene had a significant effect on the OS, PFS, and DSS of hepatocellular carcinoma patients. The prognostic indicators of survival, such as OS, PFS, and DSS, were worse in the group with high expression of the FAP gene, comparatively (*p* < 0.05) (Figures 1(d)–1(f)). Methylation analysis revealed that the FAP promoter was less methylated in the TCGA-LIHC, but the difference is not statistically significant (Figure 1(g)). Based on these results of cBioPortal, genomic alterations of FAP in TCGA-LIHC are shown in Figure 1(h). The mutation rate of FAP was only 0.8%, suggesting that mutations were not the main cause of the FAP expression changes. In conclusion, high expression of FAP in UVM tumor tissues was associated with poor patient prognosis. Reduced promoter methylation in the FAP genome may be responsible for the upregulation of FAP expression.

### 3.2. lncRNA AC007099.1 Exosomes Were Defined as Potential Contributors to the Upregulation of FAP Expression in Hepatocellular Carcinoma

**Figure 2:**
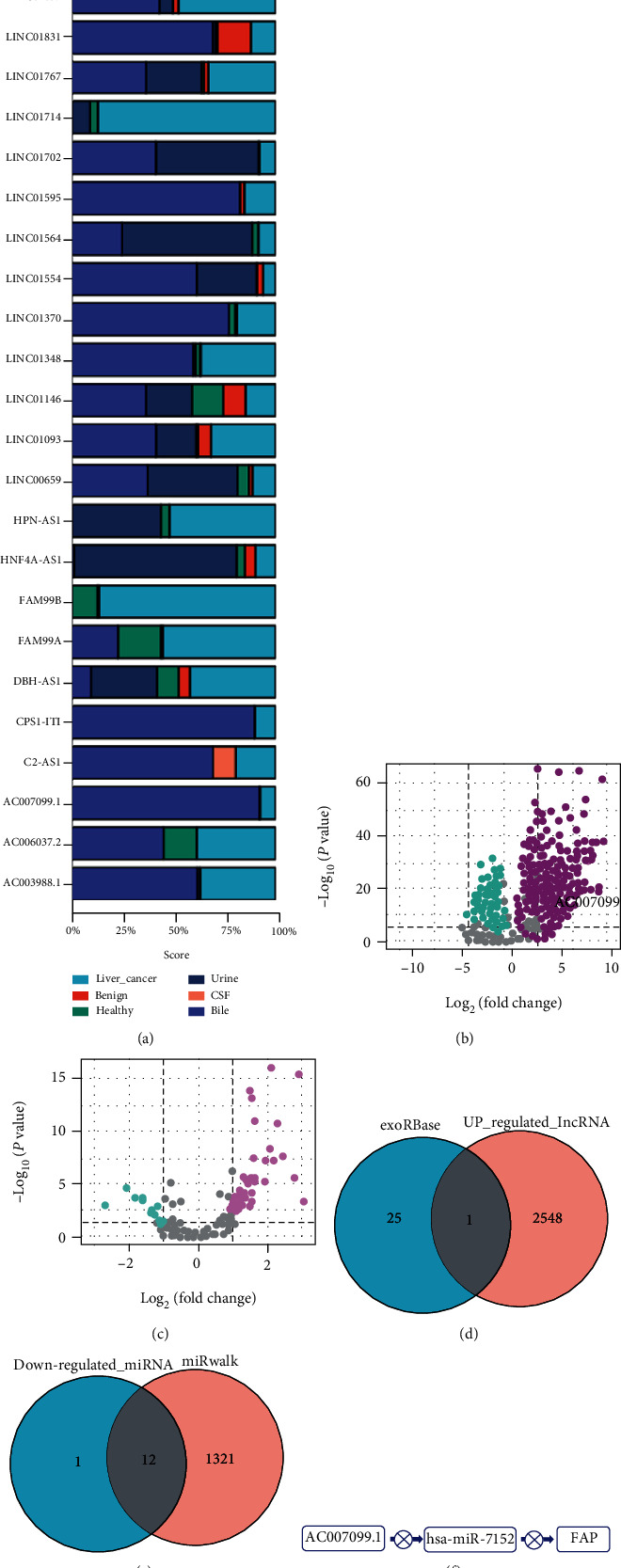
lncRNA AC007099.1 exosomes were defined as potential contributors to the upregulation of *FAP* expression in hepatocellular carcinoma. (a) A map of the distribution of all exosomes in the exoRBase liver cancer dataset. (b) Volcano map of differentially expressed mRNAs in healthy controls and liver cancer groups. (c) Volcano map of differentially expressed lncRNAs in healthy controls and liver cancer groups. (d) One of the 2549 upregulated lncRNAs in the TCGA-LIHC dataset was cooccurring in the exoRBase liver cancer exosome database (log2FC > 1). (e) The 1333 potential target genes of AC007099.1 identified in the miRWalk dataset and 12 coregulated genes among 13 downregulated miRNAs in the TCGA-LIHC dataset (log2FC < −1). (f) Constructed functional model of AC007099.1/hsa-miR-7152/*FAP* regulation.

Based on the exoRBase dataset, all detected lncRNAs in liver cancer exosomes are shown in Figure 2(a). Figure 2(a) shows that lncRNA AC007099.1 is mainly expressed in bile, suggesting that lncRNA AC007099.1 may be associated with the process of bile secretion. Volcano plots of differentially expressed mRNA and lncRNA in healthy control and liver cancer groups are shown in Figures 2(b) and 2(c). AC007099 was also present among the 2549 upregulated lncRNAs in the TCGA-LIHC datasets of the exoRBase liver cancer exosome database (Figure 2(d)). The 1333 potential target genes of AC007099 identified in the miRWalk dataset and the 13 downregulated miRNAs in the TCGA-LIHC dataset were intersected to obtain 12 common downregulated genes (log2FC < −1) (Figure 2(e)). Among them, miR-7152 was regulated by AC007099. Accordingly, we constructed a model of the AC007099/hsa-miR-7152/FAP regulatory network (Figure 2(f)).

### 3.3. Correlation Analysis and Prognostic Analysis of AC007099.1/miR-7152/FAP

**Figure 3:**
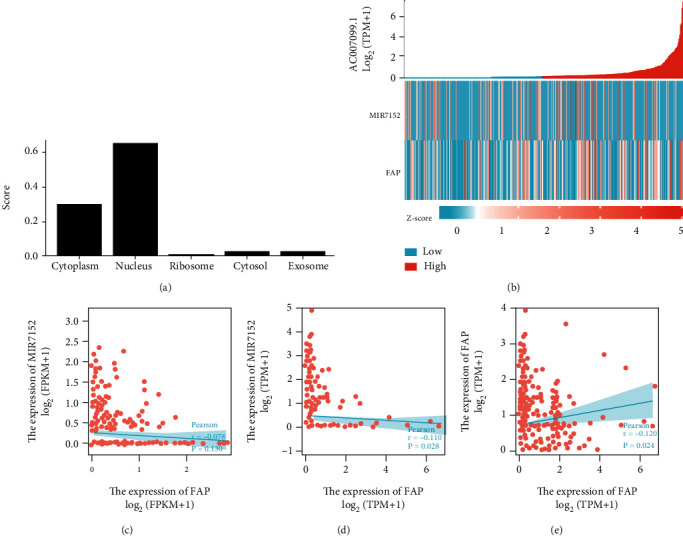
Correlation analysis of AC007099.1/miR-7152/*FAP*. (a) Expression pattern of lncRNA AC009099 distribution in subcellular. (b) Heat map of coexpressed genes of lncRNA AC009099, MIR7152, and *FAP*. (c, d) Correlation of lncRNA AC009099, MIR7152, and *FAP* was demonstrated using Person correlation analysis.

The expression pattern of lncRNA AC009099.1 in the subcellular distribution suggested that it was enriched in the cytoplasm, nucleus, cytosol, and exosome (Figure 3(a)). As predicted, AC009099.1 and FAP were negatively correlated with miR-7152 expression. AC009099.1 was positively correlated with the FAP expression (Figures 3(b)–3(d)). High expression of AC009099.1 was correlated with good OS and DSS, however, not with PFI (Figures 3(a)–3(c)). Hsa-miR-7152 and AC009099.1 were highly expressed in normal and tumor tissues, respectively (Figures 3(d)–3(f)). The ROC curves suggest that miR-7152 (AUC = 0.568), FAP (AUC = 0.813), and AC007099 (AUC = 0.732) are good predictors of hepatocarcinogenesis (Figure 3(g)).

### 3.4. AC007099.1/miR-7152/FAP Is Associated with Immune Infiltration in Patients with Hepatocellular Carcinoma

**Figure 4:**
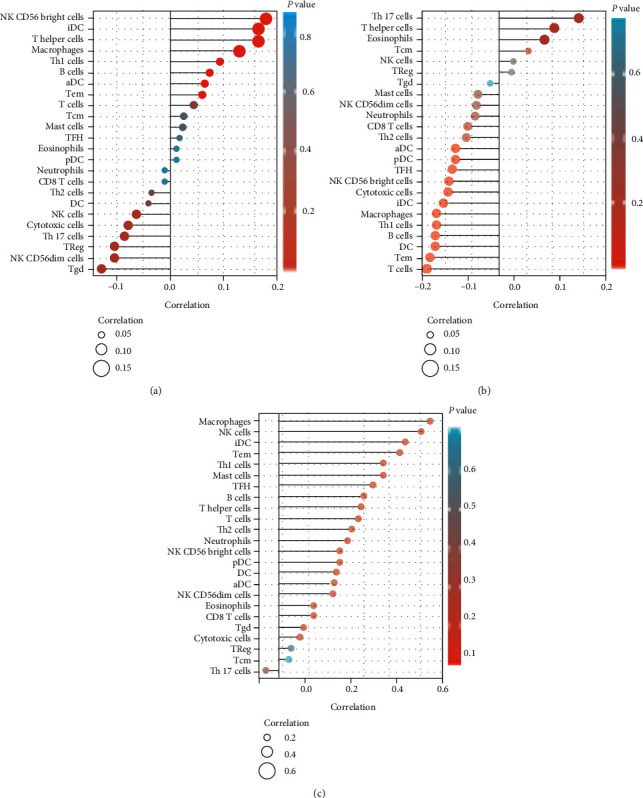
Immunological correlation analysis of AC007099.1/miR-7152/*FAP* and patients with hepatocellular carcinoma. (a) Expression of AC007099.1 in hepatocellular carcinoma correlates with immune cell infiltration. (b) Expression of miR-7152 in hepatocellular carcinoma correlates with immune cell infiltration. (c) Expression of *FAP* in hepatocellular carcinoma correlates with immune cell infiltration.

**Figure 5:**
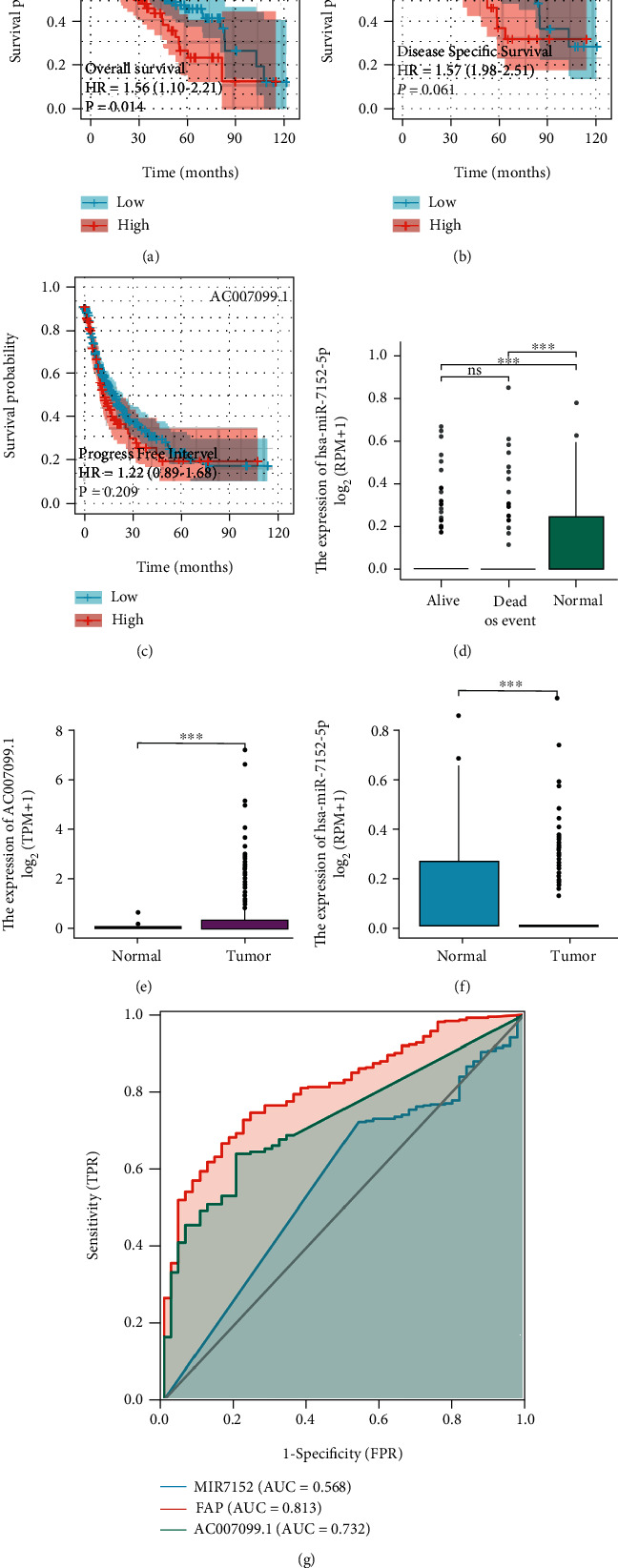
AC007099.1/miR-7152/*FAP* is associated with the prognosis of patients with hepatocellular carcinoma. (a–c) Relationship between lncRNA AC009099 and overall survival time (OS)/progression-free survival time (PFS)/disease-specific survival rate (DSS). (d) Histogram of the relationship between miR-7152-5p expression and hepatocarcinogenesis. (e) Expression of lncRNA AC009099 in hepatocellular carcinoma and normal tissues adjacent to the carcinoma. (f) Expression of miR-7152-5p in hepatocellular carcinoma and normal tissues adjacent to the carcinoma. (g) ROC curves were used to demonstrate the discriminatory ability of AC0070099, *FAP*, and miR7152 against hepatocellular carcinoma.

The AC007099.1/miR-7152/FAP pathway was found to be associated with immune infiltrations in patients with hepatocellular carcinoma. High expression of AC007099.1 was positively correlated with the infiltration of CD56 bright NK cells, iDC, and T helper cells. It was negatively correlated with the infiltration of Treg, CD56 dim NK cells, and Tgd (Figure 4(a)). High expression of miR-7152 was positively correlated with the infiltration of Th17 cells. It was negatively correlated with the infiltration of Macrophages, Th1 cells, B cells, DC, Tem, and T cells (Figure 4(b)). The high expression of FAP was positively correlated with the infiltration of most immune cells (Figure 4(c)). Therefore, the potential AC007099.1/miR-7152/FAP pathway was also found to be associated with immune infiltration in patients with hepatocellular carcinoma (Figure 5).

### 3.5. Enrichment Analysis of AC007099.1/miR-7152/FAP

**Figure 6:**
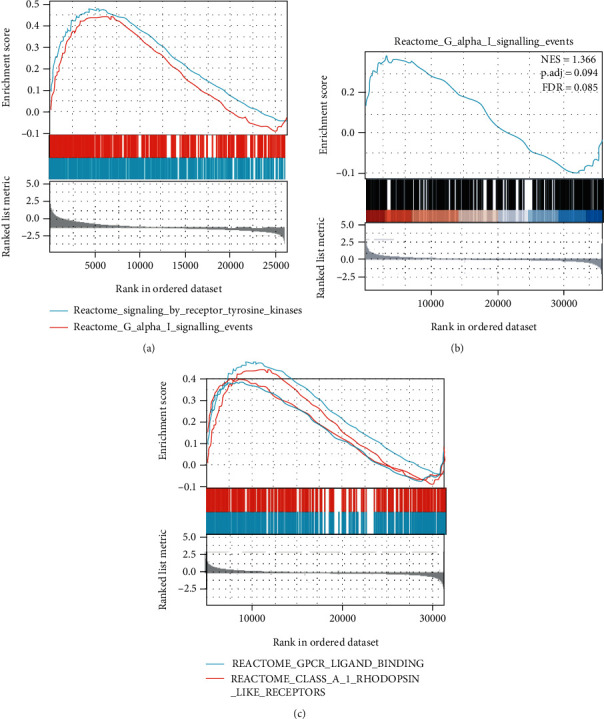
GSEA: (a) GSEA of *FAP* in hepatocellular carcinoma; (b) GSEA of miR-7152 gene in hepatocellular carcinoma; (c) GSEA of liver cancer lncRNA AC009099.

AC007099.1, miR-7152, and FAP were investigated for potential specific signaling pathways and potential molecular mechanisms (NOM *p* < 0.05, FDR *q* < 0.05). Normalized enrichment scores (NES) were calculated. GSEA results showed that AC007099.1 was enriched in GPCR ligand binding and class-A/1 rhodopsin-like receptors (Figure 6(a)). miR-7152 was enriched in G-alphaI signaling events (Figure 6(b)). FAP was enriched in signaling by receptor tyrosine kinase and G-alphaI signaling events (Figure 6(c)). The above results suggest that AC007099.1, miR-7152, and FAP may be associated with neuropeptide-related functional pathways.

## 4. Discussion

*FAP* was specifically expressed in the liver, and its low expression levels in hepatocellular carcinoma may be implicated in the poor prognosis of the disease. The AC009099/miR-7152/FAP ceRNA regulatory network was constructed in this study. The protein encoded by the human *FAP* gene is a single-channel transmembrane protein containing 760 amino acids, a short cytoplasmic amino-terminal (6 amino acids), a transmembrane region (20 amino acids), and a large extracellular domain [6, 7]. Previous studies have shown that *FAP* plays an important role in predicting tumor aggressiveness. Its frequent expression in malignant tumors suggests that FAP-targeted therapy may be a very attractive strategy [36]. Currently, much research has been devoted to FAP as a protease in the tumor microenvironment. Since FAP is expressed in most epithelial cancers and sarcomas, it has a wide range of applications in cancer [37, 38]. The survival analysis results showed that *FAP* gene expression levels significantly affected the OS, PFS, and DSS in patients with hepatocellular carcinoma. An observed high expression of FAP in UVM tumor tissues was associated with poor patient prognosis and reduced promoter methylation in the FAP gene. FAP hydrolyses a variety of neuropeptides and does serious work in maintaining normal neuroendocrine function [39]. The results of the enrichment analysis suggest that AC007099.1, miR-7152, and FAP may be associated with neuropeptide-related functional pathways. Thus, the AC009099/miR-7152/FAP ceRNA regulatory network is thought to be functionally relevant to neuropeptides.

The lncRNA AC007099.1 was found to be differentially expressed in hepatocellular carcinoma exosomes. miRNA is an important risk marker for HCC and liver injury [40, 41]. When lncRNAs and mRNAs have the same microRNA response element (MRE), there is competition between them for the same kind of miRNA. In other words, the level of lncRNA expression in the cell directly affects the number of miRNAs that can be bound by the corresponding mRNAs; that is, lncRNAs indirectly regulate the expression level of mRNAs through the bridge of MRE and thus regulate cellular functions. miR-7152 was found to be possibly regulated by AC009099. AC009099 and *FAP* expression levels were negatively correlated with miR-7152 expression, while AC009099 and *FAP* expression were positively correlated [42]. AC007099.1 and *FAP* were highly expressed while miR-7152 expression was decreased in hepatocellular carcinoma. Therefore, an AC009099/miR-7152/FAP ceRNA regulatory network was constructed in this study. Moreover, AC009099, miR-7152, and FAP were all found to be associated with infiltration of numerous immune cells in this study. The present study revealed that the expression of AC007099.1, miR-7152, and *FAP* in hepatocellular carcinoma could be used as a novel independent prognostic marker based on their observed roles during poor prognosis.

Based on their expression levels, potential targets for hepatocellular carcinoma treatment may include AC00709, FAP, and miR-7152. AC007099.1 and *FAP* were highly expressed, while miR-7152 expression was reduced in hepatocellular carcinoma. Hence, AC007099.1 may regulate the expression of miR-7152 through adsorption, thus promoting the proliferation, migration, and invasion of hepatocellular carcinoma cells. However, the bioinformatics analysis was only based on the TCGA database, which has some limitations. These hepatocellular carcinoma-related signaling pathways and key genes need further validation by various tests using molecular biological techniques and experimental data.

## 5. Conclusions

In conclusion, exploring the expression, regulatory mechanism, and prognostic value of FAP in hepatocellular carcinoma will be beneficial to the clinical treatment of patients with hepatocellular carcinoma and provide a new set of potential molecular targets for the treatment of hepatocellular carcinoma. In this study, a neuropeptide-related AC009099/miR-7152/FAP ceRNA regulatory network was constructed.

## Data Availability

The transcriptome sequencing data of the TCGA-LIHC dataset and the corresponding clinical data were downloaded from The Cancer Genome Atlas (TCGA) database. The human gene annotation file was downloaded from the Ensemble website (http://asia.ensemble.org/index).

## Abbreviations

TCGA: The Cancer Genome Atlas
FAP: Fibroblast activation protein
HPA: The Human Protein Atlas
OS: Overall survival
PFI: Progress free interval
DSS: Disease specific survival
HCC: Hepatocellular carcinomas
MRE: MicroRNA response element.
